# Right coronary artery originating from left anterior descending artery: a case report

**DOI:** 10.1186/1749-8090-5-49

**Published:** 2010-06-08

**Authors:** Hilmi Tokmakoglu, Orhan Bozoglan, Levent Ozdemir

**Affiliations:** 1 Tekden Hospital, Cardiovascular Surgery Departmant, Kocasinan-Kayseri-Turkey

## Abstract

Right Coronary Artery (RCA) originating from left anterior descending artery is a very rare congenital coronary artery anomaly. A 66-year-old man presented with hypertension and complaints of exertional chest pain. The angiography was performed. Aortic root angiography showed no coronary ostium orginating from the right sinus of valsalva. Right coronary artery was vizualized as anomalously originating from the midportion of left anterior descending artery. Severe stenosis were seen in ostium of anomalous right coronary artery, in midportion of left anterior descending and in midportion of circumflex artery. The patient was referred for coronary artery bypass grafting. The patient underwent coronary artery bypass surgery for three vessels. He was discharged home on postoperative day 7 without any complication. His echocardiogram on follow-up visit revealed good biventricular function.

## Background

Congenital coronary artery anomalies are rare and usually an incidental finding during coronary angiography. Most of them have no clinical signifance. Right Coronary Artery (RCA) originating from left anterior descending artery (LAD) is a very rare congenital coronary artery anomaly. We present a patient with three vessel disease in whom the right coronary artery originated as a seperate branch from the midportion of LAD.

## Case Report

**Figure 1:**
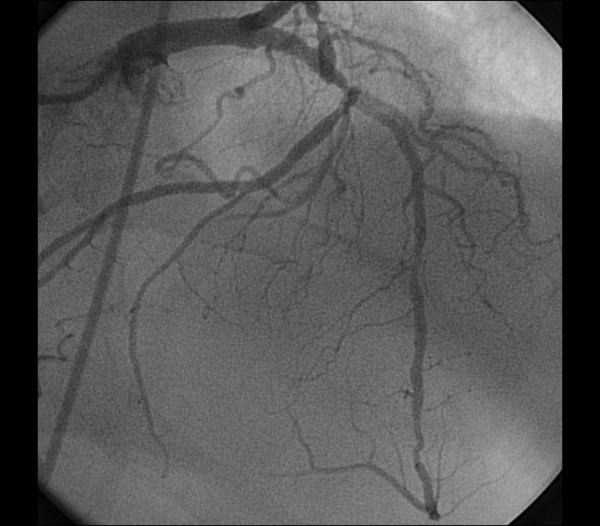
**Right Coronary Artery Originating from the midportion Left Anterior Descending Artery with coursing to the familiar area of the RCA**.

**Figure 2:**
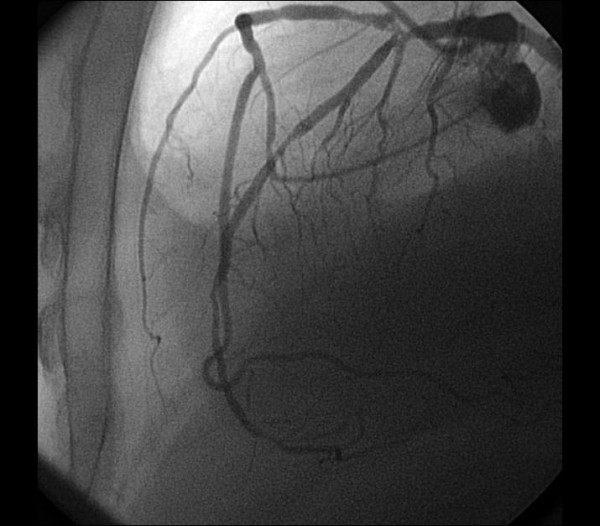
**Right Coronary Artery Originating from the midportion Left Anterior Descending Artery with coursing to the familiar area of the RCA**.

**Figure 3:**
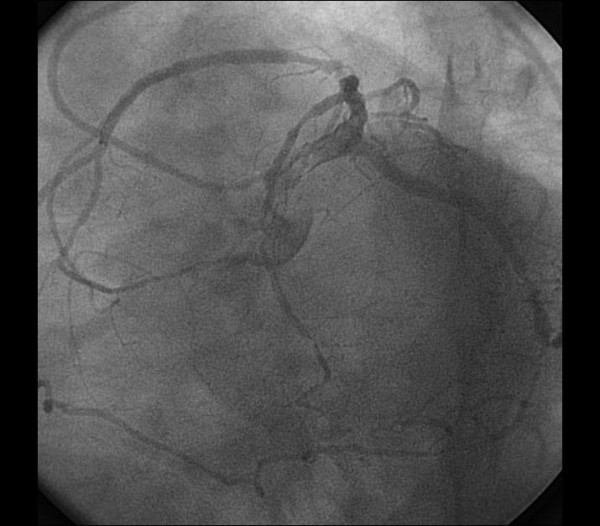
**The course of RCA in right atrioventricular groove and its continuation**.

**Figure 4:**
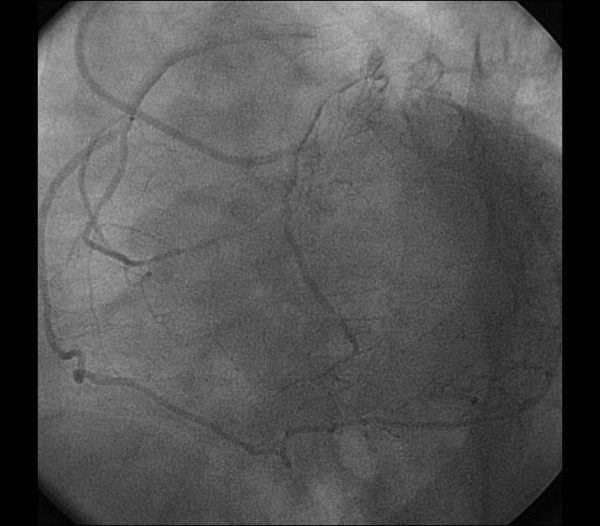
**The course of RCA in right atrioventricular groove and its continuation**.

A 66-year-old man with hypertension presented to the hospital with complaints of exertional chest pain for two months. His electrocardiogram and echocardiography were unremarkable. The angiography was performed upon persistent chest pain. During his diagnostic coronary angiogram, multiple attempts to cannulate the RCA with the right Judkins catheter were unsuccessful. Aortic root angiography showed no coronary ostium orginating from the right sinus of valsalva. RCA was vizualized as anomalously originating from the midportion of LAD artery with coursing to the familiar area (Figure 1, 2) and its continuation (Figure 3, 4). Severe stenosis were seen in ostium of anomalous RCA, in midportion of LAD and in midportion of circumflex artery. The patient was referred for coronary artery bypass grafting. The patient underwent coronary artery bypass surgery for three vessels. He was discharged home on postoperative day 7 without any complication. His echocardiogram on follow-up visit revealed good biventricular function.

## Discussion

The most common coronary anomaly is the circumflex coronary artery arising from the right sinus or the RCA, with an incidence of 0.37%-0.6% [1,2]. The next most common and pathologically significant anomalies are the right coronary artery from the left sinus of valsalva and the left main coronary artery arising anomalously from the right sinus of Valsalva. The combined incidence of these defects 0.17% in autopsy series and 0.1%-0.3% in patients undergoing catheterization or echocardiography [3-5]. A variety of anomalous origin of the RCA has been reported, including the left anterior sinus with variable courses, ascending aorta above the sinus level, descending thoracic aorta, left main coronary artery, circumflex coronary artery, the pulmonary arteries, or below the aortic valve [6-8]. Single coronary artery occupies approximately 0.024% of the general population [9]. In most of the cases, aberrant RCA originates from the left main coronary artery and traverses anterior to the right ventricle or between the pulmonary trunk and ascending aorta [10,11].

The RCA originating as a branch from the midportion of the LAD is a very rare anomaly. Six cases have been reported in the literature so far, and no patient had underlying congenital heart disease [12,13]. In our patient RCA was stemming from the midportion of the LAD and had not congenital heart disease.

Most of the coronary anomalies remain asymptomatic and are incidental to investigations by coronary angiography. Coronary artery anomalies are classified as benign (80.6%) but potentially serious anomalies (19.4%) [6]. However, myocardial perfusion can be affected, ranging from exertional angina to sudden death, within the different subtypes of these anomalies, such as a coronary artery arising from the pulmonary artery and a single coronary artery arising from either the left or right sinus of valsalva [6,10].

The pathophysiology of the restricted coronary blood flow seen in the presented case anomaly is suggested to be as follows. The acute takeoff angle, slit-like orifice, and compression of the intramural segment by the aortic valve commissure. Lateral luminal compression of the intramural portion of the coronary artery and compression of the coronary artery between aorta and pulmonary artery are also other possible ischemic mechanism [14-16]. Some autopsy-based studies have shown that slit-like orifice structure and acute angle takeoff are more common in sudden cardiac death patient [14-16]. However, there is still controversy concerning the mechanism by which the interarterial course is compressed between the aorta and pulmonary artery. An intravacular ultrasound study found that luminal compression of the coronary artery was totally attributable to the aorta because the pressure of the pulmonary artery was much lower than that of the aorta [17]. In our patient there was exertional angina. There was no surgical finding related with compression of the coronary artery between aorta and pulmonary artery.

**In conclusion**, RCA as a branch of LAD is very rare coronary anomaly. If RCA course is not between aorta and pulmonary artery, this anomaly is accepted as relatively benign rare anomaly. In case of classic appearence of RCA was not established during angiography physician should kept in mind that RCA can stem from LAD artery.

## Competing interests

The authors declare that they have no competing interests.

## Authors' contributions

HT: Chief of the Cardiovascular Surgery Departmant, performed the coronary artery bypass grafting and primary author. OB: Cardiovascular Surgeon, assisted in surgery and preparing manuscript. LO: Cardiologist, provided pre-operative care and advice during the manuscript writing process. All authors read and approved the final manuscript.

## Consent

Written informed consent was obtained from the patient for publication of this case report and any accompanying images. A copy of the written consent is available for review by the Editor-in-Chief of this journal.
